# Spatio-temporal patterns of highly pathogenic avian influenza virus subtype H5N8 spread, France, 2016 to 2017

**DOI:** 10.2807/1560-7917.ES.2018.23.26.1700791

**Published:** 2018-06-28

**Authors:** Claire Guinat, Gaëlle Nicolas, Timothée Vergne, Anne Bronner, Benoit Durand, Aurélie Courcoul, Marius Gilbert, Jean-Luc Guérin, Mathilde C. Paul

**Affiliations:** 1 École Nationale Vétérinaire de Toulouse, Toulouse, France; 2 Institut National de la Recherche Agronomique, Toulouse, France; 3 Université Libre de Bruxelles, Brussels, Belgium; 4 These authors contributed equally to this work; 5 Institut de Recherche pour le Développement, Montpellier, France; 6 Direction Générale de l’Alimentation, Paris, France; 7 Agence Nationale de Sécurité Sanitaire de l’Alimentation, Maisons-Alfort, France; 8 Fonds National de la Recherche Scientifique, Brussels, Belgium

**Keywords:** highly pathogenic avian influenza, H5N8, France, domestic poultry, spatio-temporal clustering, spread rate

## Abstract

France is one of Europe’s foremost poultry producers and the world’s fifth largest producer of poultry meat. In November 2016, highly pathogenic avian influenza (HPAI) virus subtype H5N8 emerged in poultry in the country. As of 23 March 2017, a total of 484 confirmed outbreaks were reported, with consequences on animal health and socio-economic impacts for producers.** Methods:** We examined the spatio-temporal distribution of outbreaks that occurred in France between November 2016 and March 2017, using the space–time K-function and space–time permutation model of the scan statistic test. **Results**: Most outbreaks affected duck flocks in south-west France. A significant space–time interaction of outbreaks was present at the beginning of the epidemic within a window of 8 km and 13 days. This interaction disappeared towards the epidemic end. Five spatio-temporal outbreak clusters were identified in the main poultry producing areas, moving sequentially from east to west. The average spread rate of the epidemic front wave was estimated to be 5.5 km/week. It increased from February 2017 and was negatively associated with the duck holding density. **Conclusion**: HPAI-H5N8 infections varied over time and space in France. Intense transmission events occurred at the early stages of the epidemic, followed by long-range jumps in the disease spread towards its end. Findings support strict control strategies in poultry production as well as the maintenance of high biosecurity standards for poultry holdings. Factors and mechanisms driving HPAI spread need to be further investigated.

## Introduction

Highly pathogenic avian influenza (HPAI) virus subtype H5N8 has recently resulted in major outbreaks in Europe. The emergence and spread of the disease caused by this virus has had serious consequences for animal health and a dramatic socio-economic impact for European poultry producers. Since October 2016, up to 21 European countries have experienced HPAI-H5N8 outbreaks in poultry and/or wild birds [1]. While in Germany, Romania and Switzerland the HPAI-H5N8 virus was mainly reported in wild birds, in Bulgaria, France, and Hungary it was mostly reported in poultry [1,2]. 

France is one of Europe’s foremost poultry producers and is the world’s fifth largest producer of poultry meat, behind the United States, China, Brazil and Mexico [3]. In 2015, French poultry production was estimated at over 965 million birds per year.

The first HPAI-H5N8 outbreak in poultry in France occurred on 28 November 2016. Despite the implementation of extensive control measures, including movement restrictions, establishment of 3 and 10-km radius protection and surveillance zones, stamping out of infected poultry and pre-emptive culling of poultry, the HPAI-H5N8 virus continued to spread. By 23 March 2017, the date of the last outbreak, a total of 484 HPAI-H5N8 outbreaks had been reported in poultry in the country. 

HPAI-H5N8 outbreaks reported during the 2016–17 epidemic were mostly characterised by severe clinical signs and mortality in poultry holdings [4]. This clinical pattern contrasted with what was observed during the previous HPAI outbreaks in France (2015–16), which were caused by other subtypes and for which no or only mild clinical symptoms were observed in poultry holdings [5-7]. During the 2016–17 epidemic, about 6.8 million poultry were culled as part of control measures, causing huge economic losses for the French poultry industry. Access restrictions of poultry products to international trade also severely affected French poultry stakeholders.

Most of the HPAI-H5N8 outbreaks in France since 2016, have been reported in the south-west of the country. This region is the world’s leading producer of fattening ducks, accounting for more than 70% of the world’s production and exporting ca 5,000 tons of *foie gras* per year. The sustained transmission of HPAI-H5N8 despite the extensive control measures implemented demonstrates the difficulty of controlling the spread of HPAI in the region, which is characterised by a high density of duck holdings, outdoor farming and movements of fattening ducks. As a result, HPAI is now considered one of the top priority livestock diseases in France, and improved detection and control is necessary.

A systematic understanding of how the HPAI-H5N8 outbreaks distribution varied over space and time in the poultry sector, with regard to the culling strategy implemented, is still lacking. The objective of this study was therefore to analyse the spatio-temporal distribution of outbreaks and estimate the spread rate of the front wave to provide insights into the epidemic dynamics, discuss the impact of culling strategies and inform future research directions for HPAI in poultry.

## Methods

### Data collection and management

Data on the HPAI-H5N8 outbreaks that occurred in poultry in France during the 2016–17 epidemic (November 2016–March 2017) were obtained from the Direction Générale de l’Alimentation (DGAl) of the French Ministry of Agriculture, Paris, France. An outbreak was defined as the detection of at least one laboratory-confirmed HPAI-H5N8 infected animal (by virus isolation or PCR) in a domestic poultry holding. Data included the list of laboratory-confirmed outbreaks, the species involved, the geographical locations (Cartesian coordinates) and the date of suspicion by passive or active surveillance (as further described). Outbreaks for which the precise location was missing were given the coordinates of the centroid of the commune (smallest administrative unit in France, with a median area of 10 km^2^) where they occurred. The coordinates of the communes’ centroids were obtained from GEOFLA [8]. Data on duck holding census in France was obtained from the DGAl. The data included the list of duck holdings, the geographical locations (Cartesian coordinates) and the date of culling during the 2016–17 epidemic. All geographical data were projected to RGF93/Lambert-93 (EPSG: 2154) and processed using R software version 3.3.2 [9].

### Data analysis

All of the HPAI-H5N8 outbreaks that were detected by passive surveillance (i.e. with origin of suspicion based on the appearance of clinical signs) in poultry in France during the 2016–17 epidemic (November 2016–March 2017) were included in the analyses to study how the epidemic progressed over time and space. Outbreaks identified through enhanced active surveillance of flocks were not considered. Enhanced active surveillance involved testing samples collected on flocks before pre-emptive culling, before transport between premises, when showing an epidemiological link with an outbreak and when located in the restriction zones. The reason for not including outbreaks detected by active surveillance was that this would have likely increased the detection probability of infected holdings in the vicinity of reported outbreaks and bias estimations. 

### Spatio-temporal analysis

We defined two study periods to represent two phases of the epidemic during which different control measures were applied. During the first period, from 28 November 2016 (date of the first HPAI-H5N8 outbreak) to 2 February 2017, pre-emptive culling of outdoor duck flocks within a 3-km radius circle centred on reported outbreaks was implemented in the departments of Gers, Haute-Garonne, Hautes-Pyrénées, Landes, and Pyrénées-Atlantiques. During the second period, from 3 February 2017 to 23 March 2017 (date of the last HPAI-H5N8 outbreak), country-wide measures were implemented. These included pre-emptive culling of all poultry within a 1-km radius circle centred on reported outbreaks and of all outdoor duck flocks within a 3-km radius circle (if only one outbreak was detected) or a 10-km radius circle (if several outbreaks were detected). The measures were prompted by the increasing number of outbreaks reported in the Landes department at the beginning of February 2017 [10].

Global spatio-temporal clustering of HPAI-H5N8 outbreaks detected by passive surveillance was investigated in the first and second study periods using the space–time K-function (Supplement 1) [11-13]. The space–time K-function analysis was conducted using a maximum space–time window of 30 km and 30 days. The overall significance of space–time clustering was assessed by generating 9,999 Monte Carlo random permutations. The excess risk attributable to space–time interaction within distance s and time *t* (*D_0_*(*s,t*)) was calculated and visually inspected. The analyses were performed in R software version 3.3.2 [9] using the ‘splancs’ package [14].

The presence of local spatio-temporal clusters was investigated using the space–time permutation model of the scan statistic test (Supplement 1) [15-17] implemented in the SatScan software version 9.4 [18]. The analysis was conducted across the entire epidemic period (November 2016–March 2017) to determine whether clusters could be identified during the first or the second study period. Most likely clusters were reported at a significance level of 5% based on 9,999 Monte-Carlo replications, without geographical overlap, using a maximum elliptic spatio-temporal window set to 25% of outbreaks detected by passive surveillance (i.e. around 54 outbreaks) to scan for local clusters and 25% of the study period (i.e. around four weeks).

### Spread rate analysis

The spread rate patterns of HPAI-H5N8 outbreaks detected by passive surveillance were investigated based on trend surface analysis (TSA) using the thin plate regression splines interpolation (TPRS) method and a neighbouring spread rate estimator [19,20]. A raster layer of 1 km spatial resolution was created where each pixel value represented the week of first invasion. The spread rate of the front wave of the epidemic was calculated as the inverse of the local slope of this travelling wave on the first week of a local outbreak. The value was estimated at each pixel in kilometre per week. A 12.5 km radius smoothing filter was used to avoid infinite local values for the disease spread rate. To select HPAI-H5N8 outbreaks located within the main affected areas of the 2016–17 epidemic, a Gaussian-kernel density surface with a fixed bandwidth of 15 km was calculated. The mask was created with pixels in which the smoothed density of outbreak reports was higher than one and applied to the TPRS analysis to avoid an edge effect on the estimated values. The analyses were performed in R software version 3.3.2 [9] using the ‘fields’ package [21].

To analyse the relationship between the spread rate and the duck holding density, we used the census duck holding database to calculate, for each outbreak, the number of susceptible duck holdings present within 10 km from the infected holding during the week of the outbreak (Supplement 2). A linear regression was used to assess the statistical association between the disease spread rate at the outbreak location and the density of susceptible duck holdings around outbreaks.

## Results

### Descriptive analysis

**Figure 1:**
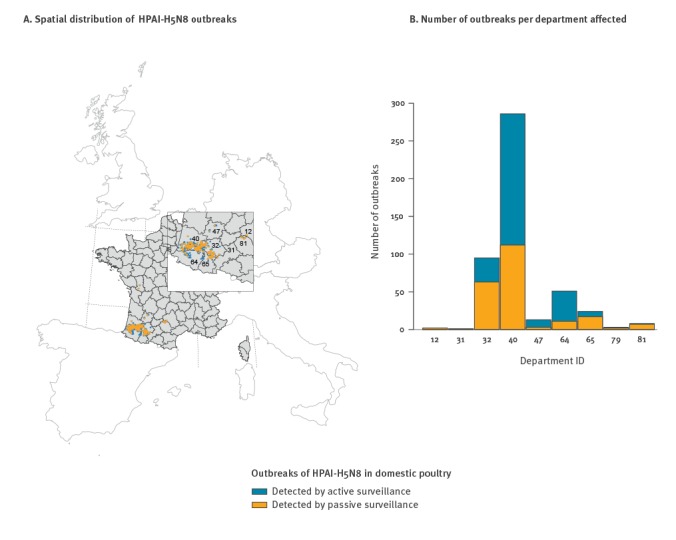
Distribution of all of HPAI-H5N8 outbreaks reported in poultry (n = 484, dark blue) including those detected by passive surveillance (n = 218, light blue), France, 28 November 2016–23 March 2017

From 28 November 2016 to 23 March 2017, 484 HPAI-H5N8 outbreaks were reported in poultry in nine departments and 235 communes. Two outbreaks without coordinates were attributed the coordinates of the centroids of the communes in which they occurred. The outbreaks were mainly located in south-west France (Figure 1A), with 59.1% (286 of 484) distributed in Landes, 19.8% (96 of 484) in Gers, 10.5% (51 of 484) in Pyrénées-Atlantiques and 5.0% (24 of 484) in Hautes-Pyrénées (Figure 1B). 

HPAI: highly pathogenic avian influenza.

Numbers on the map (panel A) and on the X axis of the chart (panel B) represent the French Departments IDs; 12: Aveyron, 31: Haute-Garonne, 32: Gers, 40: Landes, 47: Lot-et-Garonne, 64: Pyrénées-Atlantiques, 65: Hautes-Pyrénées, 79: Deux-Sèvres 81: Tarn.

**Figure 2:**
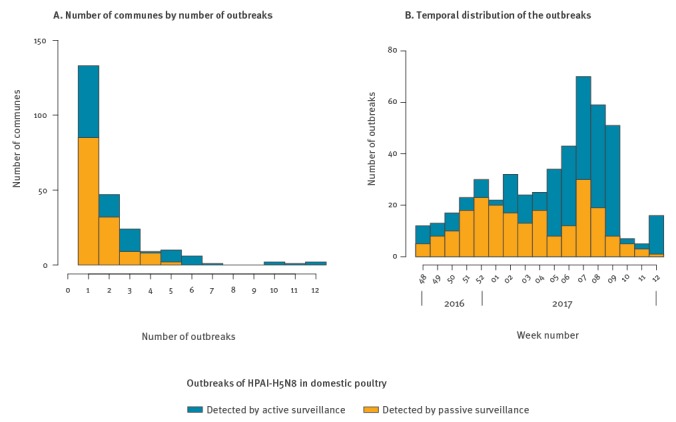
Distribution of number of HPAI-H5N8-affected communes per number of outbreaks and temporal distribution of the outbreaks, France, 28 November 2016–23 March 2017 (n = 484 outbreaks)

The number of outbreaks per commune ranged from one to 12 (Figure 2A), with 43.4% (102 of 235) of the communes experiencing more than one outbreak. The occurrence of HPAI-H5N8 varied over time (Figure 2B), with two successive peaks detected around Week 52 (starting on 26 December 2016) and Week 7 (starting on 13 February 2017). Outbreaks were mainly detected by passive surveillance (63.1%, 137/217) until the beginning of the second study period (3 February–23 March 2017), when outbreaks were mainly detected by active surveillance (69.7%, 186/267), mainly by testing samples collected on flocks during the extensive pre-emptive culling campaigns. 

HPAI: highly pathogenic avian influenza.

The majority of outbreaks affected duck flocks (81.6%, 395/484), followed by chicken (12.2%, 59/484) and multispecies flocks (5.8%, 28/484) (i.e. chickens, ducks and geese) (two outbreaks showed no species reported). Of the 484 outbreaks, 464 (95.9%) occurred in commercial poultry flocks, while the remainder 20 were in backyard flocks (4.1%) (Table 1). Concerning outbreak-affected duck holdings, information on production stages was available for 380. The majority handled all production stages (breeding and force-feeding ducks) (40.8%, 155/380), followed by holdings specialised in breeding (34.5%, 131/380) and force-feeding ducks (20.5%, 78/380) (Table 1).

**Table 1:** Distribution of HPAI-H5N8 outbreaks per type of poultry holdings, species and duck production in France, 2016–2017 epidemic (n = 484 outbreaks)

Parameter	Number of outbreaks	Total	Percentage
Type of poultry holdings in terms of commercial/backyard production			
Commercial poultry holdings	464	484	95.9
Backyard poultry holdings	20	484	4.1
Type of species			
Duck	395	484	81.6
Chicken	59	484	12.2
Multispecies	28	484	5.8
Type of duck holding in terms of production stages			
Breeding + Force-feeding	155	380^a^	40.8
Breeding	131	380^a^	34.5
Force-feeding	78	380^a^	20.5
Other	16	380^a^	4.2

^a^ Information on production stages was available only for 380 outbreaks.

### Spatio-temporal analysis

Of the 484 outbreaks, 218 were detected by passive surveillance (45.0%, Figure 1A) and used for the spatio-temporal analysis. Of these 218 outbreaks, 137 occurred during the first study period and 81 during the second.

**Figure 3:**
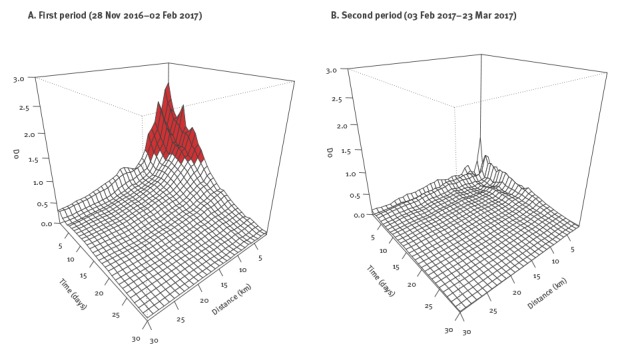
Excess risk attributable to space–time interaction (*D_0_*) as a function of space (in km) and time (in days) during two periods of the HPAI-H5N8 epidemic, 28 November 2016–23 March 2017

The global spatio-temporal clustering of the HPAI-H5N8 outbreaks in poultry was statistically significant in each study period (p < 0.05), indicated by *D_0_* values (excess risk attributable to space–time interaction) that were superior to 0. However, the intensity of the spatio-temporal interaction, varied between the two study periods (Figure 3). 

HPAI: highly pathogenic avian influenza.

The red-shaded area shows the space–time combinations for which the observed number of events was higher than twice the expected number (*D_0_* > 1).

During the first (28 November–2 February 2017), the intensity of clustering was high, with *D_0_* values exceeding 1.0 over the first 8 km and 13 days (Figure 3A). For example, *D_0_* > 1 was observed for 13 days up to 1 km, and 11 days up to 3 km. This indicates that the observed number of outbreaks which were located within 8 km around a given outbreak and, which occurred within 13 days after the date of suspicion of this given outbreak,**was greater than at least twice the number of outbreaks that would have been observed in the absence of space–time interaction (i.e. random distribution in space and time). It was also found that the intensity of clustering decreased with *D_0_* values falling below 1 at 10 km. 

Figure 3B shows that during the second study period (3 February–23 March 2017), the intensity of the spatio-temporal interaction was much smaller (*D_0_* < 1) than during the first period, even for very small space–time windows.

**Figure 4:**
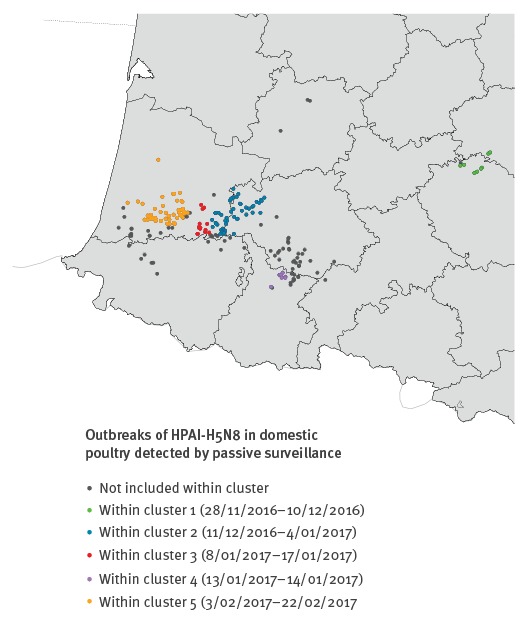
Geographical location of the most likely spatial-temporal clusters (p < 0.05) as detected by the space–time permutation scan test for 218 HPAI-H5N8 outbreaks reported in French poultry holdings, 28 November 2016–23 March 2017

The space–time permutation scan statistic test identified five statistically significant spatio-temporal clusters (p < 0.05) for which the prevalence of HPAI-H5N8 outbreaks was higher than what would be expected if outbreaks were randomly distributed (Figure 4). Figure 4 shows the geographical location of the clusters numbered according to time of occurrence. 

HPAI: highly pathogenic avian influenza.

The clusters are numbered according to time of occurrence.

Most clusters were temporarily distinct, with cluster 1 centred in an area in the north of Tarn department, cluster 2 in the west of Gers department, cluster 3 in the south-east of Landes department and cluster 5 in the south of Landes, with maximal spatial extension ranging between 16.5 and 52.7 km and maximal temporal duration ranging between 10 and 25 days (Table 2). Only cluster 4 was located in an area in the north of Hautes-Pyrénées department and showed a relatively smaller maximal spatial (11.4 km) and temporal (2 days) extension. The spatial distributions of outbreaks and clusters varied throughout the time period, showing a spatial progression of the disease spread from east to west (Figure 4). Cluster 5 was the largest cluster with maximal spatial extension of 52.7 km and temporal duration of 20 days. This cluster connected 51 of 81 outbreaks (63.0%) that occurred from 3 February onwards (Table 2), which corresponds to the beginning of the second study period (3 February–23 March 2017).

**Table 2:** Spatio-temporal clusters detected by the space–time permutation scan test for 218 HPAI-H5N8 outbreaks reported in French poultry holdings from 28 November 2016–23 March 2017

Cluster number	Cluster centre	Radius (km)^a^	Time frame	Number of outbreaks	Expected outbreaks	Observed-to-expected ratio	p value
1	Tarn	16.5**^a^**	28 Nov 2016–10 Dec 2016 (13 days)	8	0.4	21.8	0.0000
2	Gers	18.9 – 28.4**^a^**	11 Dec 2016–4 Jan 2107 (25 days)	53	16.8	2.6	0.0000
3	Landes	8.5 – 17.0**^a^**	8 Jan 2017–17 Jan 2017 (10 days)	15	1.9	7.0	0.0001
4	Hautes-Pyrénées	7.6 – 11.4**^a^**	13 Jan 2017–14 Jan 2017 (2 days)	8	0.22	22.7	0.0090
5	Landes	35.1 – 52.7**^a^**	3 Feb 2017–22 Feb 2017 (20 days)	51	13.6	3.3	0.0000

HPAI: highly pathogenic avian influenza.

^a^ The area in which cluster 1 occurred was characterised as being within a circle, while the areas of the other clusters were characterised as being inside ellipsoids.

### Spread rate analysis

**Figure 5:**
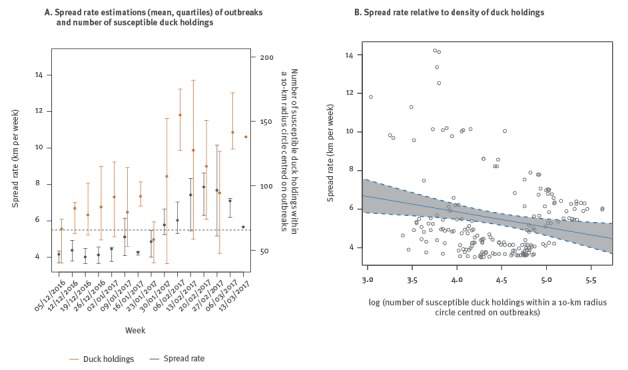
Spread rate of HPAI-H5N8 outbreaks in poultry holdings in relation with number and density of duck holdings, France, 28 November 2016–23 March 2017 (n = 192 outbreaks)

Of the 218 outbreaks detected by passive surveillance, 192 (88.1%) were located within the density surface and selected for the spread rate analysis. The estimated spread rate of the front wave of HPAI-H5N8 disease from December 2016 to March 2017 averaged 5.5 km/week. Around 7% of the outbreaks (13 of 192) showed an estimated average spread rate that was higher than 10 km/week. During the first study period, the average spread rate was mainly below 5.5 km/week but during the second study period (3 February–23 March 2017), it was above 5.5 km/week, with a peak value of 7.9 km/week (Week 8 starting on 20 February 2017) (Figure 5A).

The dashed grey line in panel A indicates the estimated spread rate averaged over the 192 HPAI-H5N8 outbreaks. The dates (day/month/year) on the X axis of the chart indicate the beginning of each week considered. In panel B, the blue line indicates the fitted regression line and the dashed blue lines the confidence intervals.

Across the study period, the average number of susceptible duck holdings within 10 km from the outbreaks ranged from 58.2 (Week 4, starting on 23 January 2017) to 155.2 (Week 6, starting on 06 February 2017). As shown in Figure 5A, the density of susceptible duck holdings around outbreaks as well as the spread rate at the outbreak location increased significantly over time (p < 0.05). An increased estimated spread rate at the outbreak location was significantly associated with a decreased density of susceptible duck holdings around outbreaks (p = 0.005, slope = -0.8) (Figure 5B).

## Discussion

This study explored the spatio-temporal patterns of HPAI-H5N8 spread over a 4-month period following the introduction of the virus into France in November 2016. Most of the outbreaks were reported in fattening duck holdings located in south-west France (Landes and Gers departments) during the winter months (December 2016–February 2017). In winter 2015, these populations had already shown susceptibility to HPAI infection, with up to 80 outbreaks of HPAI virus of subtypes H5N1, H5N2 and H5N9 reported between November 2015 and August 2016 [5,7]. In Europe, countries with high density of duck holdings were also affected by HPAI, particularly those with duck holdings that could not sufficiently be protected against contacts with wild birds [1]. In France, most of the outbreaks occurred in commercial poultry holdings but the lack of census data on backyard poultry holdings impedes any quantitative comparison with this sector.

Through the space–time K function analysis, the intense space–time interaction (*D_0_* > 1) observed during the first study period (November 2016–early February 2017), suggested that outbreaks were at least twice more likely to occur within a short period of time (for up to 13 days) and distance (under 8 km), indicating the presence of localised transmission processes (without excluding the possibility of long-distance dispersal events). This is consistent with previous evidence on higher transmission rates of HPAI in the vicinity of infected poultry holdings [22]. Decreases in space–time interaction intensity (1 > *D_0_* > 0) during the second study period (early February–March 2017) indicated more evidence for a scattered propagation with potential long-range jumps in the disease dispersal. This could be related with the decrease in the number of susceptible poultry holdings around outbreaks, that resulted from the implementation of pre-emptive culling measures during the second study period (Supplement 2). Using the space–time permutation model, four clusters were identified during the first period and covered smaller geographical areas and shorter time periods than those of the sole cluster identified during the second period. This is in line with the more intense disease transmission process observed in the first period compared with the second period in the space–time K function analysis. The absence of clusters regarding the remaining 69 outbreaks (grey dots in Figure 4) indicates that these outbreaks tended to be more sporadically distributed over the study period. They also showed a tendency to spread over time from eastern to western parts of south-west France, where the highest densities of fattening duck holdings are reported in the country. One should note that the direction of disease spread could be influenced by prevailing winds, as was shown in the Netherlands during the 2003 HPAI epidemic in this country [23,24], but this has not been investigated yet in the French context.

This study generated the first estimates of the front wave velocity of HPAI-H5N8 disease. Across the whole period and all of the affected departments, the estimated spread rates averaged 5.5 km/week. The velocity of the HPAI-H5N8 spread was relatively homogeneous during the first study period at around 4 km/week but increased up to 7.9 km/week during the second period. This supports that the disease spread over short and long distances in the first and second periods, respectively, which is consistent with the distribution of outbreaks observed in the previous analyses. The increase in the spread rate during the second study period suggests that reducing the density of poultry holdings around outbreaks did not prevent the appearance of long-distance transmission events and had a limited effect on the propagation speed of the disease. In line with the spatial extent of the clusters, the spread rate analysis suggests that HPAI-H5N8 can spread over distances larger than the radius of the surveillance zone currently used (10 km), highlighting the necessity to conduct further research on the optimal ring culling radius for controlling the HPAI epidemics [25,26]. The study also demonstrated that the density of duck holdings influenced the velocity of HPAI-H5N8 spread. Although seemingly counter-intuitive, results show that lowest densities were associated with the highest spread rates, suggesting that even a spatial scattered distribution of duck holdings and low densities were favourable to HPAI-H5N8 spread. The increase of the spread rate in those areas may be due to some long-distance jumps, which could be primarily explained by transport of ducks between holdings (over short or large distances) which are frequent in fattening duck production. While regulations prevented any movements of poultry from outside and within the restricted zone, movements of people, vehicles and equipment could still occur [10]. However, further risk factor analyses are needed to test these hypotheses. In addition to poultry movements, the presence of wild birds in the vicinity of poultry holdings during the epidemic period has also been suggested as a possible route of disease transmission [27,28]. As of 23 March 2017, 52 outbreaks of HPAI-H5N8 had been reported in wild bird species (mostly anatids, but including also larids, columbids and falconids) mainly in south-western and eastern areas of France [29], but the potential range and rate of distance dispersal of HPAI by wild birds remains unclear.

The findings of this study may have been influenced by a number of elements. First, the sensitivity of passive surveillance might not have been optimal, rendering it impossible to ascertain the status of poultry holdings where HPAI-H5N8 presence was not reported. However, the HPAI-H5N8 outbreaks in the 2016–17 epidemic in France were characterised by infections with severe clinical signs of both in chickens and ducks affecting a large proportion of the flocks. Given the severity of the clinical signs, the risk of unreported clinical cases can therefore be considered relatively low [1]. Second, the analysis refers to the date of suspicion, i.e. the date at which the first clinical signs were observed, as the date of disease introduction into the poultry holding. While this is a continuous pattern in the diagnosis, this might slightly influence the outcomes of the study since the incubation period ranges roughly from 1 to 5 days at the individual level (and could be longer at the flock level due to the variation between animals), although its estimation is difficult and can hardly be extrapolated from experimental infections [30]. Another limitation of the study is that spread rate estimations at the spatial and temporal edge of the epidemic should be interpreted with caution as the interpolation of first time of invasion is supported by fewer points in those areas, and may somewhat bias the results.

This study provides insights into the 2016–17 French HPAI epidemic dynamics. The epidemic was mostly characterised by intense transmission events at the early stages, followed by long-range jumps in the disease dispersal towards the end. Findings support the implementation of strict control strategies targeted at poultry production, such as culling of infected and suspected flocks, and local movement restrictions. They also support the need to maintain high biosecurity standards on poultry holdings. There is a need for further research on evaluating the optimal culling level, the risk factors for transmission between holdings and the role of poultry movements combined with phylogenetics to help understand the HPAI-H5N8 transmission patterns.
